# l-arginine, asymmetric and symmetric dimethylarginine for early outcome prediction in unselected cardiac arrest victims: a prospective cohort study

**DOI:** 10.1007/s11739-021-02767-z

**Published:** 2021-06-03

**Authors:** Beata Csiszar, Zsolt Marton, Janos Riba, Peter Csecsei, Lajos Nagy, Kalman Toth, Robert Halmosi, Barbara Sandor, Peter Kenyeres, Tihamer Molnar

**Affiliations:** 1 Division of Cardiology, 1st Department of Medicine, Medical School, University of Pécs, Pécs, Hungary; 2 Szentagothai Research Centre, University of Pécs, Pécs, Hungary; 3 Department of Neurosurgery, Medical School, University of Pécs, Pécs, Hungary; 4 Department of Applied Chemistry, University of Debrecen, Debrecen, Hungary; 5 Department of Anaesthesiology and Intensive Therapy, Medical School, University of Pécs, Pécs, Hungary

**Keywords:** Cardiopulmonary resuscitation, Post-resuscitation care, Asymmetric dimethylarginine, Prognostication, Cardiac arrest, Mortality

## Abstract

Early prediction of the mortality, neurological outcome is clinically essential after successful cardiopulmonary resuscitation. To find a prognostic marker among unselected cardiac arrest survivors, we aimed to evaluate the alterations of the l-arginine pathway molecules in the early post-resuscitation care. We prospectively enrolled adult patients after successfully resuscitated in- or out-of-hospital cardiac arrest. Blood samples were drawn within 6, 24, and 72 post-cardiac arrest hours to measure asymmetric and symmetric dimethylarginine (ADMA and SDMA) and l-arginine plasma concentrations. We recorded Sequential Organ Failure Assessment, Simplified Acute Physiology Score, and Cerebral Performance Category scores. Endpoints were 72 h, intensive care unit, and 30-day mortality. Among 54 enrolled patients [median age: 67 (61–78) years, 48% male], the initial ADMA levels were significantly elevated in those who died within 72 h [0.88 (0.64–0.97) µmol/L vs. 0.55 (0.45–0.69) µmol/L, *p* = 0.001]. Based on receiver operator characteristic analysis (AUC = 0.723; *p* = 0.005) of initial ADMA for poor neurological outcome, the best cutoff was determined as > 0.65 µmol/L (sensitivity = 66.7%; specificity = 81.5%), while for 72 h mortality (AUC = 0.789; *p* = 0.001) as > 0.81 µmol/L (sensitivity = 71.0%; specificity = 87.5%). Based on multivariate analysis, initial ADMA (OR = 1.8 per 0.1 µmol/L increment; *p* = 0.002) was an independent predictor for 72 h mortality. Increased initial ADMA predicts 72 h mortality and poor neurological outcome among unselected cardiac arrest victims.

**Supplementary Information:**

The online version contains supplementary material available at 10.1007/s11739-021-02767-z.

## Introduction

Management of post-resuscitation care, including post-cardiac arrest syndrome, ischemic brain injury, myocardial dysfunction, and multiple organ failure (MOF) remains an unmet clinical challenge with high mortality. The 1-year survival rate after out-of-hospital cardiac arrest (OHCA) is around 8% [1] and 13% among in-hospital cardiac arrest (IHCA) patients [2]. Early and effective prediction of the mortality, neurological and functional consequences is clinically essential for the medical team to choose the optimal level of treatment, to decide about withdrawal of life-sustaining therapy, to guide goals-of-care conversations with relatives, and to improve the cost-effectiveness of care [3]. Current guidelines recommend a multimodal approach (neurological examination, electrophysiological investigations, neuroimaging, and biomarkers) for prognostication of neurological outcome after resuscitation from cardiac arrest [4]. The major limitation of the current algorithm is that it can be applied only to a minority of patients who remain comatose. To improve the quality of prognostication after cardiac arrest, addressing the role of extracerebral causes of death is warranted [5]. Only one-fourth of patients who suffered IHCA die due to neurological injury, while most of them may reach acceptable neurological function but suffer from MOF which may lead to death [6]. A reliable biomarker that can be used in unselected resuscitated patients would provide useful information about the general outcome and survival without focusing only on the neurological status.

Endothelial injury, microcirculatory dysfunction, and coagulopathy developing after resuscitated cardiac arrest are associated with poor outcome [7]. The nitric oxide system plays a crucial role in the regulation of vascular tone and nitric oxide is identified as a mediator of vasodilation under a variety of physiological and pathophysiological conditions, such as hypoxia and ischemia [8]. Enzymatic activities of nitric oxide synthases catalyze the two-step oxidation of l-arginine to nitric oxide and l-citrulline. Methylarginines are described as the main regulators and endogenous inhibitors of nitric oxide synthase catalytic function by competing with l-arginine for binding to the catalytic site of nitric oxide synthase (NG-monomethyl l-arginine and asymmetric dimethylarginine—ADMA) or by binding to the l-arginine membrane uptake carrier (ADMA and symmetric dimethylarginine—SDMA). ADMA has been demonstrated to inhibit nitric oxide formation and increase oxidative stress in vascular endothelial and smooth muscle cells [9].

Previous investigations found a strong association of high plasma ADMA level upon admission and mortality in critically ill patients [10]. Elevated circulating concentrations of l-arginine derivatives have been associated with progression and outcome in a variety of conditions including cardiovascular [11] and cerebrovascular disorders [12–14].

We aimed to explore, for the first time, the alterations of the l-arginine-nitric oxide pathway molecules in the early post-resuscitation care of cardiac arrest survivors, and their distinct association patterns with the prognostic scoring systems, neurological function, and outcome measures such as 72 h, intensive care unit (ICU) and 30-day mortality.

## Materials and methods

### Study population

This is a prospective, single-center observational study conducted from January 2018 to January 2019 in the Intensive care unit of the 1st Department of Medicine, Department of Anaesthesiology and Intensive Care and Department of Emergency Medicine at the University of Pécs. Our cohort was made up of 54 adult patients [median age: 67 (61–78) years, 48% male] who suffered IHCA or OHCA, and after successful resuscitation were admitted to the ICU for post-resuscitation care. Successful resuscitation was defined as the return of spontaneous circulation (ROSC). 23 patients admitted to the ICU of the 1st Department of Medicine, 18 patients to the Department of Anaesthesiology and Intensive Care, and 13 patients from the Department of Emergency Medicine were enrolled in our cohort. Standard post-resuscitation care was applied for each patient in the ICU without interaction with the research team. Therapeutic hypothermia was not applied during post-resuscitation care, but each patient was kept in normothermia. This report follows the STROBE Statement [15]. The study was approved by the Local Ethics Committee of the University of Pécs (file number: 6941 – PTE 2018.) and has followed the principles outlined in the Declaration of Helsinki for all human investigations. Informed consent for being included in the study was obtained from legal representatives or, in case the patients had regained consciousness, from the patients themselves.

### Sample and data collection

Data collected included patient anamnestic information, comorbidities, the circumstances of cardiopulmonary resuscitation, variables that are necessary for calculating Simplified Acute Physiology Score (SAPS II) and Sequential Organ Failure Assessment (SOFA) severity scores. Mortality by 72 h after cardiac arrest, mortality occurred in the ICU and 30-day mortality, and the best neurological status was used as outcome measures. Plasma samples were collected within 6, 24, and 72 h after ROSC to determine the biomarker concentrations by high-performance liquid chromatography. Besides, laboratory and vital parameters were also assessed in the mentioned investigated time points after cardiac arrest. The SAPS II and SOFA scores were calculated according to the worst parameters of the first 24 h after cardiac arrest. The neurological outcome was measured using cerebral performance category (CPC) score, which consists of a scale of 5 levels: (1) a return to normal cerebral function and normal living, (2) disability but sufficient function for independent activities of daily living, (3) severe disability, limited cognition, inability to carry out independent existence, (4) coma and (5) brain death. CPC scores 1–3 were determined as good and 4–5 as poor neurological outcome. The best neurological status reached in the ICU was recorded using the CPC scale to avoid false pessimistic neurological classification in patients who regained consciousness after resuscitation and then died due to extracerebral causes with satisfactory neurological status during the follow-up period [16].

### Biomarkers

Blood samples were drawn into Vacutainer^®^ EDTA-tubes from resuscitated patients on admission within 6 h and 24 ± 3 and 72 ± 3 h after cardiac arrest to determine plasma concentrations of l-arginine, ADMA and SDMA. The samples were centrifuged within 10 min at 3500 rpm for 15 min. The supernatant was immediately stored in aliquot at − 80 °C until determining the l-arginine derivative concentrations at the end of the recruitment process. l-arginine, ADMA, and SDMA were measured in the plasma by high-performance liquid chromatography after derivatisation in collaboration with the Department of Applied Chemistry at the University of Debrecen, Hungary [17, 18]. We calculated the change of the investigated markers from 6 to 24 and 24–72 h. All samples were processed by the same technicians using the same equipment and blinded to all clinical data. The biomarker values were not available for clinical purposes and did not influence therapeutic approaches or the decision-making process.

### Statistical analysis

Statistical analysis of the collected data was evaluated by IBM SPSS Statistics^®^ 27.0. The Kolmogorov–Smirnov test was applied to test for normality of continuous variable distribution. Comparisons of continuous non-normally distributed data between groups were carried out using the Mann–Whitney *U* test. Student *T* test was used for analysis of normally distributed continuous data. The continuous variables are reported as medians and interquartile ranges or mean and standard deviation. Correlation analysis was performed calculating Spearman’s correlation coefficient (rho). For variables with significant correlation, linear logistic regression analysis was performed, and *R*^2^ values were reported on the figures. Receiver Operating Characteristic (ROC) analysis and the Area Under the Curve (AUC) were used to determine the most appropriate cutoff values of initial circulating ADMA levels and the investigated endpoints, especially for the evaluation of the 72 h mortality and neurological outcome based on CPC categories. Univariable binary logistic regression tests were used to evaluate associations between the recorded initial variables and 72 h mortality with corresponding beta values and 95% confidence intervals. Variables with *p* value ≤ 0.05 in the univariable analysis were included in the multivariable models considering the principle of multicollinearity. Multivariable logistic regression was used to identify factors independently associated with 72 h mortality. Sample size and power analysis were performed for the overall population using PS program version 3.1.2. For the sample size of *n* = 54, patients needed to detect a true difference of *d* = 0.267 in initial ADMA for 72 h mortality with 92% power, where type I error probability is *α* = 0.05. A *p* value < 0.05 was considered statistically significant.

## Results

**Fig. 1:**
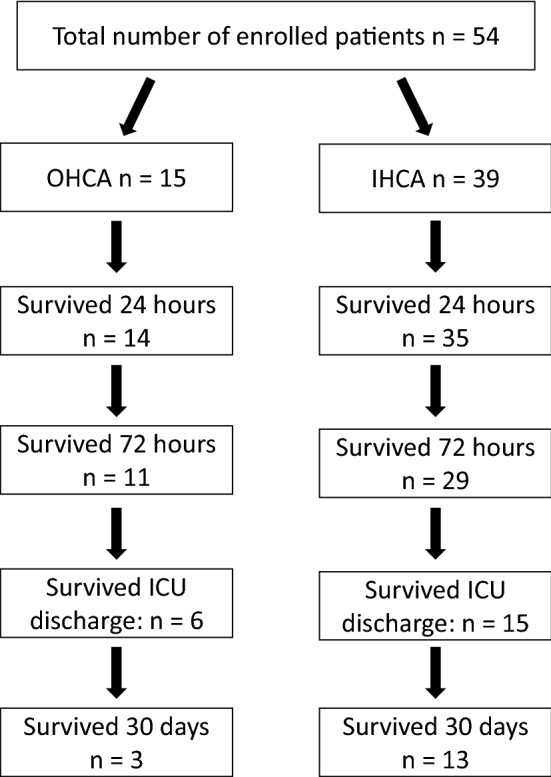
Study population: Flow-chart about the exact number of survivors (*IHCA* in-hospital cardiac arrest, *OHCA* out-of-hospital cardiac arrest, *ICU* intensive care unit)

During the 30-day follow-up, 11% of the patients reached good neurological status (CPC 1–2) and 39% had severe neurological disability (CPC 3), while half of the patients suffered from coma, vegetative state, or brain death (CPC 4–5). The biomarker levels and their change were also analyzed according to the chosen clinical endpoints. The primary endpoint was the 72 h mortality, besides, we analyzed ICU mortality, 30-day mortality and neurological outcome (CPC). Figure 1 shows the exact number of survivors during the 30-day follow-up.

### 72 hour mortality

Characteristics of the study group made up of 54 successfully resuscitated patients are summarized in Table 1. Around one-fourth of the patients died within the first 3 days after the ROSC. There was no statistically significant difference between survivors and non-survivors regarding age, gender, cardiac arrest characteristics (e.g., in-hospital or out-of-hospital, length of the cardiopulmonary resuscitation, and first monitored rhythm), suggested etiology of cardiac arrest, vital parameters on enrollment, or comorbidities. 72 h non-survivors had significantly higher points of SAPS II score.

**Table 1:** Characteristics of the study population according to 72 h mortality

	Survivors (*n* = 40; 74%)	Non-survivors (*n* = 14; 26%)	*p* value
Baseline			
Age (years)	66 [59–78]	72 [63–81]	0.309
Male gender	21 (53%)	5 (36%)	0.279
Characteristics of the CA and the CPR			
Localisation: in-hospital CA	29 (73%)	10 (71%)	0.939
Resuscitation during nightshift or weekend	28 (70%)	11 (79%)	0.538
First monitored rhythm			
Ventricular tachycardia/fibrillation	9 (23%)	5 (36%)	0.332
Pulseless electrical activity	11 (28%)	3 (21%)	0.655
Asystole	18 (45%)	5 (36%)	0.545
Unknown	2 (5%)	1 (7%)	0.762
Time of the resuscitation (min)	10 [5–24]	8 [5–19]	0.842
Patients required epinephrine	32 (80%)	13 (93%)	0.451
Dose of epinephrine (mg)	2 [1–3]	2 [1–3]	0.493
Mechanical ventilation within 6 h after CA	37 (93%)	12 (86%)	0.451
Etiology of CA			
Ischemic heart disease	12 (30%)	5 (36%)	0.692
Heart failure	13 (33%)	3 (21%)	0.435
Sepsis	3 (8%)	2 (14%)	0.451
Hyperkalaemia	3 (8%)	2 (14%)	0.451
Aspiration	3 (8%)	0	0.292
Hypothermia	2 (5%)	0	0.394
Stroke	1 (3%)	1 (7%)	0.429
Pulmonary embolism	1 (3%)	1 (7%)	0.429
Pneumonia	2 (5%)	0	0.394
Unknown	14 (35%)	3 (21%)	0.347
Parameters on enrolment			
Systolic blood pressure (mmHg)	115 [103–140]	113 [95–126]	0.667
Diastolic blood pressure (mmHg)	61 [53–68]	62 [57–69]	0.928
Mean arterial pressure (mmHg)	77 [70–91]	76 [71–84]	0.671
Heart rate (/min)	78 [65–99]	94 [79–101]	0.241
Body temperature (°C)	36.3 ± 1.3	36.2 ± 1.5	0.762
Comorbidities, previous medical history			
Hypertension	29 (73%)	10 (71%)	0.939
Ischemic heart disease	13 (33%)	7 (50%)	0.243
Diabetes mellitus	19 (48%)	3 (21%)	0.088
Heart failure	14 (35%)	3 (21%)	0.347
Permanent atrial fibrillation	8 (20%)	2 (14%)	0.636
Stroke or transient ischemic attack	8 (20%)	2 (14%)	0.636
Carotid artery stenosis	4 (10%)	1 (7%)	0.751
Chronic obstructive pulmonary disease	8 (20%)	0	0.070
Peripheral artery disease	5 (13%)	2 (14%)	0.864
Previous pulmonary embolism	2 (5%)	1 (7%)	0.763
Previous, cured malignant disease	5 (13%)	3 (21%)	0.418
Active malignant or hematologic disease	7 (18%)	2 (14%)	0.781
Prognostic scores			
SOFA	10 ± 3	12 ± 3	0.267
SAPS II	70 ± 16	87 ± 11	< 0.001

Continuous data are presented as median values with interquartile range [percentiles 25–75] or mean ± standard deviation, categorical data as the number of subjects and percentages

*CA* cardiac arrest, *CPR* cardiopulmonary resuscitation, *SOFA* Sequential Organ Failure Assessment Score, *SAPS II* Simplified Acute Physiology Score II, *ICU* intensive care unit

Table 2 summarizes the absolute plasma levels and the changes of l-arginine, ADMA, and SDMA between the patients who survived the 72 h after the cardiac arrest or died in this period. Significantly higher initial ADMA levels were observed among patients who died within 3 days. Comparing the initial ADMA levels between the IHCA and OHCA groups, we did not observe significant difference [IHCA: 0.61 (0.46–0.85) vs. OHCA: 0.64 (0.45–0.87), *p* = 0.977].

**Table 2:** L-Arginine pathway molecules and their change according to the 72 h mortality

	Survivors (*n* = 40; 74%)	Non-survivors (*n* = 14; 26%)	*p* value
Biomarker plasma levels within 6 h after CA			
L-arginine (µmol/L)	33.45 [27.84–46.96]	46.16 [27.89–72.44]	0.079
ADMA (µmol/L)	0.55 [0.45–0.69]	0.88 [0.64–0.97]	0.001
SDMA (µmol/L)	0.93 [0.65–1.60]	0.93 [0.76–1.29]	0.969
Biomarker plasma levels 24 h after CA			
L-arginine (µmol/L)	38.95 [31.26–60.56]	45.62 [17.64–70.11]	0.910
ADMA (µmol/L)	0.54 [0.45–0.78]	0.78 [0.51–1.05]	0.145
SDMA (µmol/L)	1.03 [0.75–1.98]	1.32 [0.88–2.28]	0.515
Change in biomarker plasma levels from 6 to 24 h after CA			
ΔL-arginine (24–6 h) (µmol/L)	5.16 [− 4.48 to 23.37]	− 5.21 [− 25.32 to 21.38]	0.234
ΔADMA (24–6 h) (µmol/L)	0.03 [− 0.08 to 0.10]	− 0.12 [− 0.20 to 0.02]	0.079
ΔSDMA (24–6 h) (µmol/L)	0.17 [− 0.02 to 0.42]	0.22 [0.02–0.42]	0.713

Data are presented as median values with interquartile range [percentiles 25–75]

*ADMA* asymmetric dimethylarginine, *CA* cardiac arrest, *SDMA* symmetric dimethylarginine

### ICU mortality

A total of 33 patients died in the ICU (by average 6; min. 1–max. 26 days). Investigating the ICU mortality, none of the l-arginine pathway molecules showed a significant difference between survivors or non-survivors. The ADMA levels tended to remain higher among ICU non-survivors but the difference did not reach significance. The plasma ADMA levels of ICU non-survivors decreased from 6 to 24 h, while the values of the surviving group elevated by 24 h [− 0.08 (− 0.16 to 0.05) µmol/L vs. 0.07 (− 0.04 to 0.11) µmol/L, *p* = 0.024] (Suppl.-Table 1). Subgroup analysis of IHCA patients revealed significantly decreased 6 h l-arginine/ADMA ratio in patients who died in the ICU (Suppl.-Fig. 1).

### 30-day mortality

**Fig. 2:**
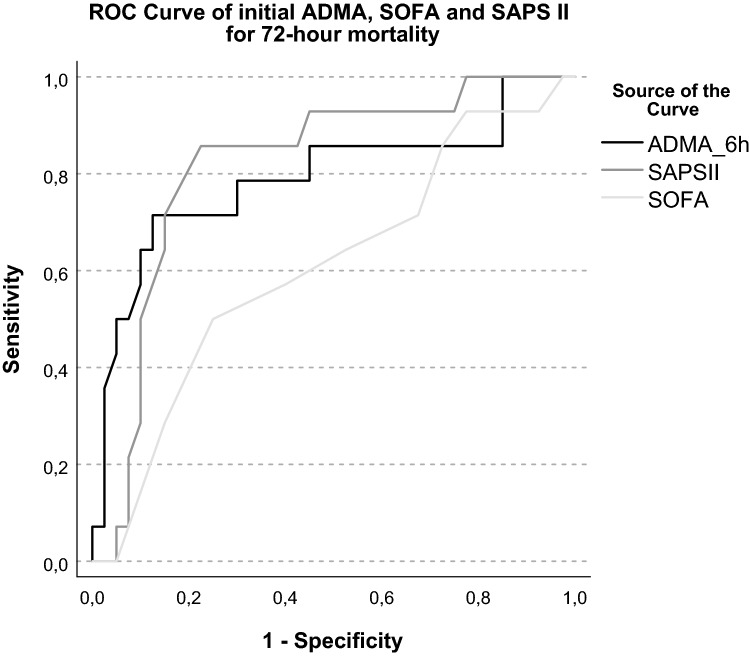
ROC Curve of initial ADMA, SOFA, and SAPS II for 72-day mortality. SAPS II AUC: 0.817 [0.688–0.946], *p* < 0.001; ADMA AUC: 0.789 [0.628–0.950], *p* = 0.001; SOFA AUC: 0.608 [0.433–0.783], *p* = 0.232 (*ADMA* asymmetric dimethylarginine, *AUC* Area Under the Curve, *SAPS* Simplified Acute Physiology Score, *SOFA* Sequential Organ Failure Assessment, *ROC* Receiver Operating Characteristic)

70% of the patients died within 30 days after cardiac arrest. Analyzing the kinetics of the markers according to 30-day mortality outcome, an opposite change was observed in ADMA level from 6 to 24 h between survivors and non-survivors similarly to the observation according to ICU mortality [− 0.08 (− 0.16 to 0.06) in non-survivors vs. 0.07 (− 0.03 to 0.11) in survivors, *p* = 0.028] (Suppl.-Table 2). In contrast, l-arginine, SDMA levels, or their change showed no significant difference in any of the clinical endpoints at any investigated time point. The l-arginine/ADMA ratio slightly elevated up to 72 post-cardiac arrest hours in the total population regardless of the mortality (6 h: 66.04 ± 4.33; 24 h: 80.04 ± 5.35; 72 h: 99.99 ± 7.13; *p* < 0.05) (Suppl.-Fig. 2).

### Prognostic scores and biomarkers

**Fig. 3:**
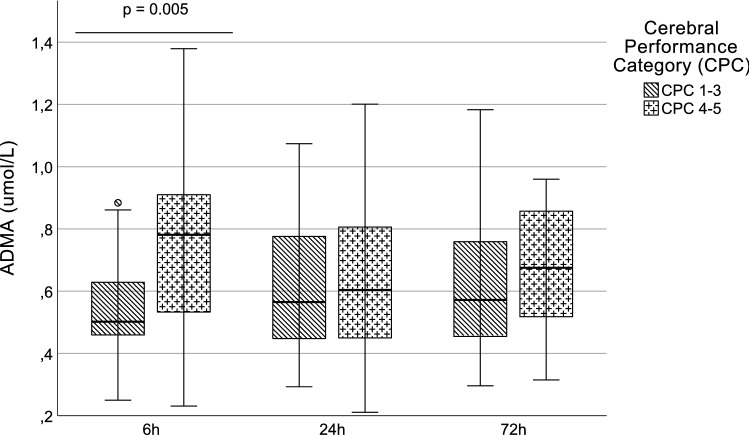
ADMA and neurological outcome (max. CPC). (*ADMA* asymmetric dimethylarginine, *CPC* cerebral performance category)

The SAPS II and SOFA score had significant but moderate prognostic value for ICU mortality based on ROC analysis [SOFA AUC: 0.695 (0.537–0.853) *p* = 0.020; SAPS II AUC: 0.747 (0.602–0.891), *p* = 0.003]. Neither SAPS II nor SOFA score showed significant difference between IHCA and OHCA subgroups. The statistical analysis revealed a significant positive correlation between the initial ADMA levels and the SAPS II score (rho = 0.393, *R*^2^ = 0.178, *p* = 0.002) (Suppl.-Fig. 3). The l-arginine levels per se did not show significant correlation with the investigated scores or parameters. Figure 2 demonstrates the analysis of the three parameters (SOFA, SAPS II, and initial ADMA) for 72 h mortality in a combined ROC curve. The results showed that the AUC of SAPS II and initial ADMA were comparable reflecting similar sensitivity and specificity in prediction of 72 h mortality, in contrast SOFA provided poor prognostic information for mortality [SAPS II AUC: 0.817 (0.688–0.946), *p* < 0.001; ADMA AUC: 0.789 (0.628–0.950), *p* = 0.001; SOFA AUC: 0.608 (0.433–0.783), *p* = 0.232].

### Neurological outcome

The initial ADMA levels were significantly elevated among patients with poor neurological outcome (CPC 4–5) (Fig. 3). ROC analysis of initial ADMA for prediction of a coma, vegetative state, or brain death (CPC 4–5) showed an AUC of 0.723 (95% CI 0.574–0.871; *p* = 0.005). Based on the ROC analysis, the best cutoff for poor neurological outcome (CPC 4–5) was determined as > 0.65 µmol/L (sensitivity: 66.7%; specificity: 81.5%).

### Independent prediction of 72 h mortality

Based on ROC analysis, initial ADMA level was found to be a predictor of 72 h mortality (Fig. 2) with a best cutoff value of > 0.81 µmol/L (sensitivity: 71.0%; specificity: 87.5%). Univariable logistic regression analyses including each variable assessed within 6 h after cardiac arrest identified initial ADMA, serum bicarbonate, and lactate levels as significant markers for 72 h mortality. Multivariable analysis revealed that initial ADMA (OR: 1.8 per 0.1 µmol/L increase in ADMA; 95% CI 1.252–2.611; *p* = 0.002) is an independent predictor for 72 h outcome after cardiac arrest (Table 3).

**Table 3.** Univariable (a) and multivariable (b) regression analysis for 72 h mortality

a. Univariable logistic regression analysis for 72 h mortality		
Variable	Odds ratio—Exp(B) (lower CI–upper CI)	*p*
ADMA 6 h (per 0.1 µmol/L increase)	1.81 (1.25–2.61)	0.002
HCO_3_^−^ 6 h	0.89 (0.79–0.99)	0.034
Lactate 6 h	1.26 (1.06–1.49)	0.008

b. Binary logistic regression analysis for 72 h mortality			
Model 1—Binary Logistic Regression—Enter	*B*	Odds ratio—Exp(B) (lower CI–upper CI)	*p*
ADMA 6 h (per 0.1 µmol/L increase)	0.573	1.77 (1.23–2.56)	0.002
HCO_3_^−^ 6 h	− 0.132	0.88 (0.77–1.00)	0.054

Model 2—Binary Logistic Regression—Enter	B	Odds ratio—Exp(B) (lower CI–upper CI)	p
ADMA 6 h (per 0.1 µmol/L increase)	0.488	1.63 (1.14–2.33)	0.008
Lactate 6 h	0.189	1.21 (0.99–1.48)	0.065

*ADMA* asymmetric dimethylarginine, *CI* confidence interval

## Discussion

To the best of our knowledge, the l-arginine pathway molecules and their change in the early post-resuscitation phase have not yet been evaluated in unselected cardiac arrest patients. Here, we investigated the prognostic value of l-arginine, ADMA, SDMA plasma levels, and kinetics in combination with other conventionally used laboratory parameters among a general, unselected population of cardiac arrest survivors including IHCA and OHCA patients. The major result of our study was the observation that initial ADMA level measured within 6 h after cardiac arrest was an independent predictor of short-term mortality and poor neurological outcome. While the 6 h ADMA levels had unequivocal prognostic value for 72 h mortality, the levels measured at either 24 and 72 h or their change did not associate with any of the investigated endpoints. Neither ICU nor 30-day mortality was predicted by any of the l-arginine pathway molecules. It was described previously that cardiovascular failure and hemodynamical instability are responsible for early death within 3 days after cardiac arrest, while later death is mainly related to neuronal injury due to severe hypoxic-ischemic encephalopathy and the subsequent withdrawal of life-sustaining therapy [19]. The different pathophysiological backgrounds of early and later post-resuscitation death may explain that ADMA, as a prognostic marker associated with the severity and mortality of many cardiovascular diseases, may also be promising for predicting mortality in the early phase of post-resuscitation care [20]. Accordingly, we could not prove the prognostic value of SDMA and l-arginine for mortality in the post-resuscitation phase.

A most recent review about the metabolism of ADMA in hypoxia summarizes the results of both animal and human studies concerning the metabolites of l-arginine pathway in various hypoxic conditions [21]. They mention more studies where ADMA levels were elevated in hypoxia, while SDMA levels did not show significant elevation. One of them found continuous increase of ADMA but not of SDMA in chronic-intermittent hypobaric hypoxia [22]. Others observed higher ADMA serum concentrations in patients with obstructive sleep apnea syndrome [23]. Furthermore, the l-arginine pathway molecules have been suggested as prognostic markers for acute exacerbation of chronic obstructive pulmonary disease [24].

Previous publications investigated the l-arginine pathway molecules in ischemic stroke [13, 25]. Plasma ADMA levels were higher in acute ischemic stroke patients compared to the control group and stroke outcome was worse in patients with increased ADMA levels than those with stable ADMA levels. They explained the observation with the mechanism that increased ADMA levels inhibit the production of endothelial nitric oxide (which plays a crucial role as an endogenous regulator of vasodilation in cerebral arterioles) consequently reducing the cerebral perfusion [25]. Other investigations also confirmed that ADMA increases the vascular tone in cerebral blood vessels and leads to cerebral hypoperfusion [26]. Molnar et al. observed that metabolites of the l-arginine pathway were elevated in the very acute phase of ischemic stroke indicating a more pronounced endothelial dysfunction compared with asymptomatic significant carotid stenosis or healthy subjects. They suggested that the elevated initial ADMA levels could be linked to the pathogenesis of endothelial cell dysfunction or could be the consequence of oxidative stress [13].

We observed a decrease from higher initial levels of ADMA levels up to 24 h among patients who died within 30 days after cardiac arrest, while ADMA levels slightly elevated in survivors. The discrepancy between the initial ADMA levels of non-survivors and survivors disappeared by 24 post-cardiac arrest hours. ADMA per se might contribute to brain injury by either reducing cerebral blood flow and facilitating excitotoxic neuronal death or contributing to the activation of the thrombo-inflammatory cascade [12]. Excessively high initial ADMA may adversely affect cerebral perfusion after cardiac arrest, leading in the short term to exacerbation of hypoxic-ischemic injury following resuscitation and early death in the post-resuscitation phase. On the other hand, elevated initial ADMA levels might indicate a more severe hypoxic insult or pre-existing endothelial dysfunction as discussed in the publication of Molnar et al. mentioned above. In the literature, only a few studies are investigating these marker levels in acute hypoxic conditions in humans. In healthy male volunteers, the mean plasma nitric oxide concentration was elevated after acute hypoxic exposure, which was associated with a reduction in plasma ADMA level leading to elevated plasma nitric oxide concentrations [27]. This finding suggests that the decrease in ADMA concentration observed on the first day after cardiac arrest in our more severe group of patients who died within 30 days may be an adaptive mechanism presumably counteracting cerebral hypoperfusion. Although it is important to note that the concentration of nitric oxide was not determined in our present study.

We observed a continuous increase of l-arginine/ADMA ratio up to 72 post-cardiac arrest hours in the total study population and found a significantly decreased l-arginine/ADMA ratio at 6 post-cardiac arrest hours in patients who died in the ICU after IHCA. These findings are consistent with the observation of Molnar et al., who suggested that a temporary increase of l-arginine along with a decrease of ADMA could be a protective mechanism after ischemic stroke [13]. Nitric oxide, a pleiotropic molecule, has several intracellular effects leading to vasorelaxation, endothelial regeneration, inhibition of leukocyte chemotaxis, and platelet adhesion [28]. In turn, lack of l-arginine, the source of nitric oxide, could lead to oxidative stress induced by hypoxic insults such as cardiac arrest, thus it may explain the reduced initial l-arginine/ADMA ratio in IHCA non-survivors. A most recent prospective observational study found that a higher arginine and lower arginine/ADMA ratio measured within 24 h after OHCA were independently associated with 90-day mortality [29]. Similarly to their findings, we could detect significantly elevated l-arginine/ADMA ratio in survivors in the IHCA group. They did not find significant difference between survivors and non-survivors regarding ADMA levels. Despite their findings, l-arginine was not a prognosticator of death in our population. Moreover, this marker did not show difference between survivors and non-survivors for none of the investigated endpoints. However, their population was exclusively made up of patients who suffered OHCA, while we enrolled mostly IHCA patients. Importantly, the pathophysiology and the most common conditions leading to death during post-resuscitation care could be different. Two-thirds of OHCA patients die due to brain injury in ICU, while MOF is the main cause of mortality after IHCA [6].

In this study, we recorded the best CPC reached in the ICU. It is generally observed, that despite patients reach acceptable neurological function during their ICU stay, they may later die as the consequence of MOF, especially after IHCA [6]. Considering this, we felt it confusing to categorize patients emerging from a comatose state into the unfavorable CPC group based on late-developing MOF. Therefore, the pure neurological outcome (regaining the consciousness) and the overall outcome (ICU or total mortality) should be separately analyzed. Therefore, the best achieved CPC score was recorded during the ICU stay similar to the study of Fabio Silvio Taccone and colleagues [16]. Importantly, eight patients (14.8% of the total population) in the CPC 1–3 group died due to non-neurological reasons in the ICU despite acceptable neurological status (death after awakening). All these eight patients had sepsis and failure of two or more organ systems.

The reliability of SOFA and SAPS II scores for prognostication in cardiac arrest patients remains unclear [30]. In our population, these scores had moderate prognostic value and we confirmed their association with l-arginine pathway molecules. Previous research findings revealed correlations among ADMA levels, l-arginine/ADMA ratio and microvascular reactivity, the extent of organ failure, and mortality in patients with sepsis [31]. In critically ill patients, ADMA correlated with SOFA score [10]. These observations are consistent with our results. Based on our findings, the prognostic accuracy of SAPS II (a time-consuming assessment method) and initial ADMA for 72 h mortality after ROSC were comparable. We conclude that early determination of initial ADMA after ROSC may be as effective and accurate as SAPS II in prediction of the early post-CPR mortality.

The strength of our study is its prospective nature using serial sampling in the first 3 days after cardiac arrest to evaluate the kinetics and changes of the l-arginine pathway molecules. Our study population was made up of unselected resuscitated patients including in- and out-of-hospital cardiac arrest survivors of three different intensive care units allowing us to explore reliable predictive markers regardless of the circumstances and etiology of cardiac arrest and covering the widest range of resuscitated patients with different comorbidities and etiology. While most studies evaluated prognostication of OHCA patients, here, we were able to find potential prognostic markers in IHCA patients and markers which might be also used in both groups. The best neurological status reached in the intensive care unit was recorded to avoid false pessimistic neurological estimation of patients (especially after in-hospital cardiac arrest) reaching acceptable neurological status but dying as the consequence of hemodynamic dysfunction or MOF.

The limitation of our study is the low total number of enrolled patients. Concerning the high mortality rate, we could not collect enough data for long-term analysis. Further research is warranted to explore the prognostic value of multiple markers (including the l-arginine pathway molecules) for long-term outcome after cardiac arrest. Probably, the no-flow time (i.e., the interval between collapse and start of CPR) would be more valuable information compared to the length of the CPR until ROSC. However, the exact time of the collapse was not known in the majority of IHCA and some OHCA patients, thus it prevented us from reporting the accurate no-flow time in this cohort. To satisfy the proportional odds assumption, we reclassified the CPC as follows: CPC 4–5 (vegetative state or death) as poor neurological outcome, and CPC 1–3 as good/moderate neurological outcome, because of the low number of individuals with really good outcome (CPC 1–2) until 30-day follow-up. The reason of the extension of the acceptable neurological outcome categories with CPC 3 was the potential for a later neurological improvement of some patients during rehabilitation. The study was conducted in a single-center, so local treatment strategies and guidelines could limit the generalisability of our findings.

## Conclusions

Here, we investigated for the first time the prognostic value and the kinetics of the l-arginine-pathway molecules during the early phase of successful cardiopulmonary resuscitation. Our results suggest that initial circulating ADMA level may indicate more severe hypoxic insult and can predict 72 h mortality among cardiac arrest victims.

## Supplementary Information

Below is the link to the electronic supplementary material.

Supplementary file1 (PDF 332 KB)

## Data Availability

All data relevant to the study are included in the article or uploaded as supplementary material. No additional data are available due to data protection requirements.
