# Supplementary material for: l-arginine, asymmetric and symmetric dimethylarginine for early outcome prediction in unselected cardiac arrest victims: a prospective cohort study
Source: Intern Emerg Med. 2021 Jun 3;17(2):525–34. doi: 10.1007/s11739-021-02767-z (PMC8964544; doi:10.1007/s11739-021-02767-z)
Supplement: Supplementary file 1 — Supplementary file1 (PDF 332 KB) [file 11739_2021_2767_MOESM1_ESM.pdf]

SUPPLEMENT MATERIAL

L-arginine, asymmetric and symmetric dimethylarginine for early outcome prediction in unselected cardiac arrest victims: a prospective cohort study.

Journal name: **Internal and Emergency Medicine**

Beata Csiszar[1,2], Zsolt Marton, Ph.D.[1,2], Janos Riba[1], Peter Csecsei, Ph.D.[3], Lajos Nagy, Ph.D.[4], Kalman Toth, D.Sc.[1,2], Robert Halmosi, D.Sc.[1,2], Barbara Sandor, Ph.D.[1,2], Peter Kenyeres, Ph.D.*[1,2], Tihamer Molnar, Ph.D.[5]

[1]University of Pecs, Medical School, 1st Department of Medicine, Division of Cardiology, Pecs, Hungary
[2]University of Pecs, Szentagothai Research Centre, Pecs, Hungary
[3]University of Pecs, Medical School, Department of Neurosurgery, Pecs, Hungary
[4]University of Debrecen, Department of Applied Chemistry, Debrecen, Hungary
[5]University of Pecs, Medical School, Department of Anaesthesiology and Intensive Therapy, Pecs, Hungary

***Author for correspondence:**
Peter Kenyeres, MD, Ph.D.
University of Pecs, Medical School, 1st Department of Medicine, Division of Cardiology
7624 Pecs, 13 Ifjusag Street, Hungary
Phone: +3672/536-000 – 32638
Fax: +3672/536-148
ORCID: https://orcid.org/0000-0002-9074-2280
E-Mail: kenyeres.peter@pte.hu

Table 1. - L-arginine pathway molecules and their change according to the ICU mortality

	Survivors (n=21; 39%)	Non-survivors (n=33; 61%)	p value
**Biomarker plasma levels within 6 hours after CA**			
L-arginin (µmol/L)	33.17 [29.43 – 52.68]	42.25 [23.59 – 49.96]	N.S.
ADMA (µmol/L)	0.58 [0.46 – 0.66]	0.70 [0.46 – 0.89]	N.S.
SDMA (µmol/L)	0.77 [0.62 – 1.73]	0.96 [0.81 – 1.46]	N.S.
**Biomarker plasma levels 24 hours after CA**			
L-arginin (µmol/L)	48.82 [31.04 – 69.06]	38.14 [23.24 – 56.77]	N.S.
ADMA (µmol/L)	0.59 [0.51 – 0.79]	0.54 [0.41 – 0.81]	N.S.
SDMA (µmol/L)	0.97 [0.74 – 2.79]	1.30 [0.85 – 1.81]	N.S.
**Biomarker plasma levels 72 hours after CA**			
L-arginin (µmol/L)	61.01 [47.35 – 77.33]	53.78 [35.98 – 75.31]	N.S.
ADMA (µmol/L)	0.60 [0.50 – 0.76]	0.62 [0.45 – 0.85]	N.S.
SDMA (µmol/L)	0.90 [0.67 – 2.20]	1.48 [0.92 – 1.75]	N.S.
ΔL-arginin (24h-6h) (µmol/L)	10.81 [-2.78 – 25.16]	2.84 [-17.13 – 13.24]	N.S.
ΔADMA (24h-6h) (µmol/L)	0.07 [-0,04 – 0.11]	-0.08 [-0.16 – 0.05]	**0.024**
ΔSDMA (24h-6h) (µmol/L)	0.21 [0.003 – 0.46]	0.11 [-0.01 – 0.39]	N.S.
ΔL-arginin (72h-24h) (µmol/L)	18.98 [-6.31 – 29.75]	8.95 [-9.21 – 28.66]	N.S.
ΔADMA (72h-24h) (µmol/L)	0.01 [-0.06 – 0.14]	0.07 [-0.06 – 0.14]	N.S.
ΔSDMA (72h-24h) (µmol/L)	-0.06 [-0.37 – 0.06]	0.16 [-0.23 – 0.55]	N.S.

Data are presented as median values with interquartile range [percentiles 25–75]. ADMA: asymmetric dimethylarginine; CA: cardiac arrest; SDMA: symmetric dimethylarginine.

Table 2. – L-arginine pathway molecules and their change according to the 30-day mortality

	Survivors (n=16; 29.63%)	Non-survivors (n=38; 70.37%)	p value
**Biomarker plasma levels within 6 hours after CA**			
L-arginin (μmol/L)	33.31 [29.24 – 51.86]	41.67 [25.01 – 51.68]	N.S.
ADMA (μmol/L)	0.54 [0.46 – 0.64]	0.67 [0.44 – 0.89]	N.S.
SDMA (μmol/L)	0.77 [0.62 – 1.96]	0.95 [0.76 – 1.43]	N.S.
**Biomarker plasma levels 24 hours after CA**			
L-arginin (μmol/L)	42.97 [28.61 – 69.06]	39.09 [31.26 – 57.75]	N.S.
ADMA (μmol/L)	0.59 [0.51 – 0.77]	0.54 [0.44 – 0.83]	N.S.
SDMA (μmol/L)	0.89 [0.7138 – 3.06]	1.18 [0.89 – 1.81]	N.S.
**Biomarker plasma levels 72 hours after CA**			
L-arginin (μmol/L)	62.32 [54.14 – 74.25]	49.26 [36.01 – 78.26]	N.S.
ADMA (μmol/L)	0.59 [0.51 – 0.79]	0.65 [0.45 – 0.84]	N.S.
SDMA (μmol/L)	1.22 [0.72 – 2.34]	1.32 [0.74 – 1.71]	N.S.
**Change in biomarker plasma levels**			
ΔL-arginin (24h-6h) (μmol/L)	10.81 [-2.48 – 26.59]	2.84 [-10.41 – 17.60]	N.S.
ΔADMA (24h-6h) (μmol/L)	0.07 [-0.03 – 0.11]	-0.08 [-0.16 – 0.06]	**0.028**
ΔSDMA (24h-6h) (μmol/L)	0.17 [0.003 – 0.56]	0.14 [-0.01 – 0.41]	N.S.
ΔL-arginin (72h-24h) (μmol/L)	19.16 [4.96 – 34.88]	8.62 [-7.60 – 27.12]	N.S.
ΔADMA (72h-24h) (μmol/L)	0.04 [-0.05 – 0.16]	0.06 [-0.07 – 0.13]	N.S.
ΔSDMA (72h-24h) (μmol/L)	-0.06 [-0.32 – 0.13]	0.08 [-0.32 – 0.47]	N.S.

Data are presented as median values with interquartile range [percentiles 25–75]. ADMA: asymmetric dimethylarginine; CA: cardiac arrest; SDMA: symmetric dimethylarginine.

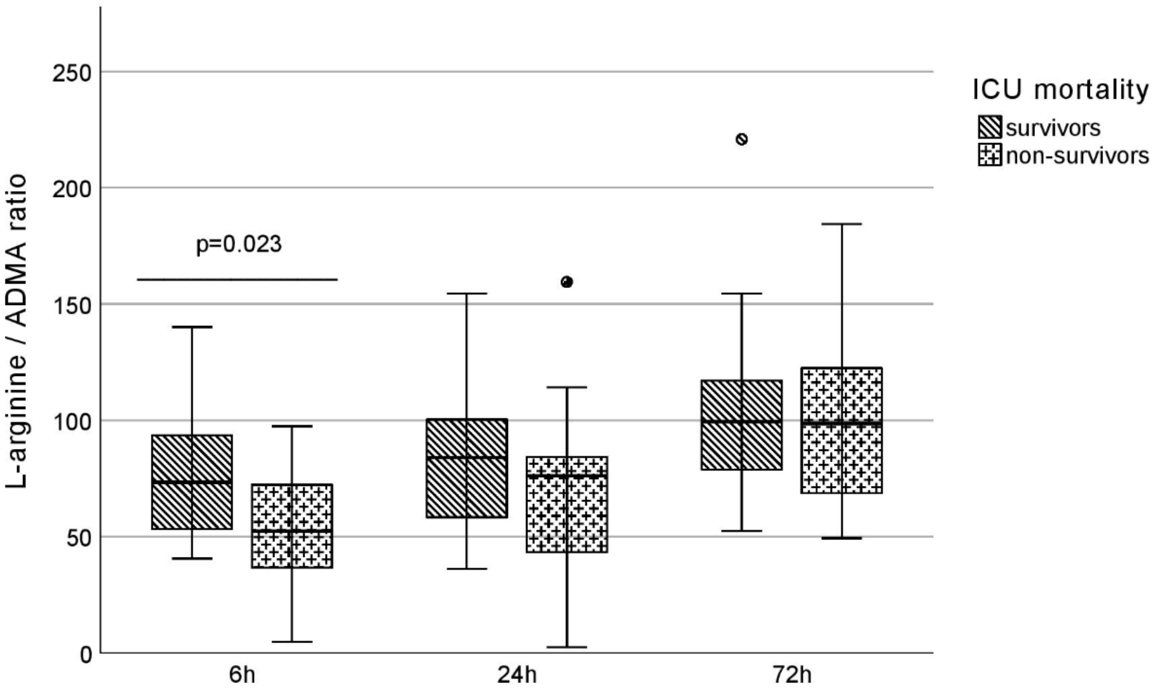

Figure 1. L-arginine/ADMA ratio and ICU mortality in IHCA group

(ADMA: asymmetric dimethylarginine; ICU: intensive care unit; IHCA: in-hospital cardiac arrest)

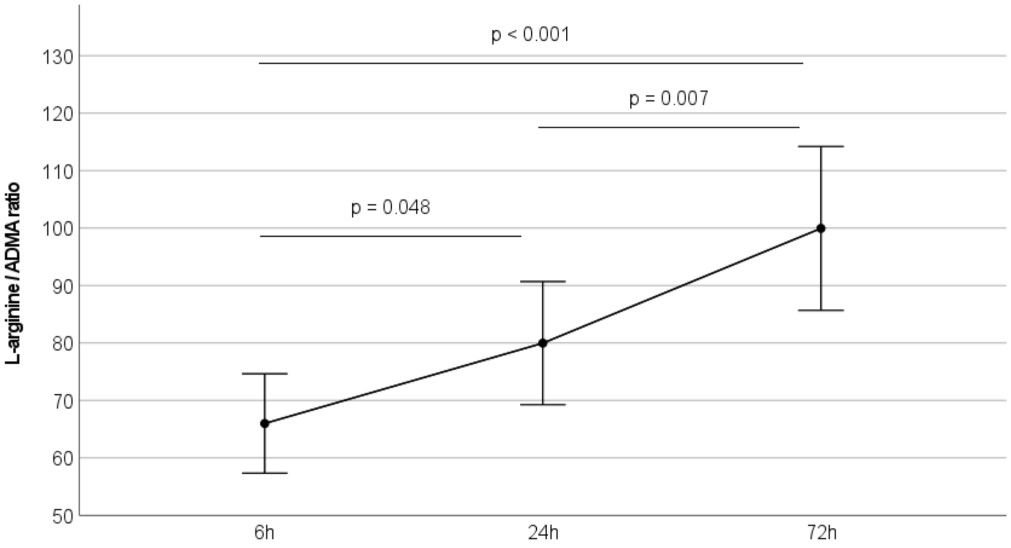

Figure 2. The kinetics of the L-arginine/ADMA ratio in the total population

(ADMA: asymmetric dimethylarginine)

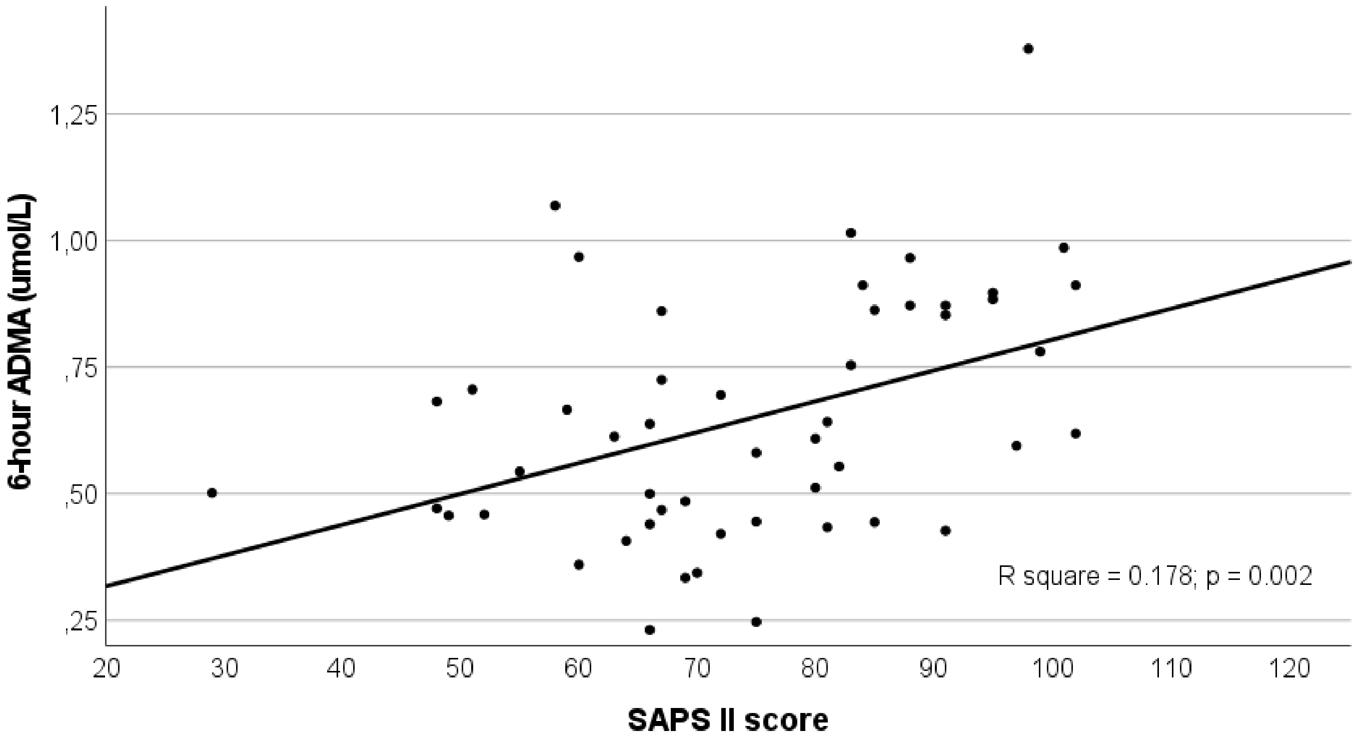

Figure 3. – Correlation between 6-hour ADMA levels and SAPS II score

(ADMA: asymmetric dimethylarginine, SAPS: simplified acute physiology score)
